# A Systematic Review of Barriers to Breast Cancer Screening, and of Interventions Designed to Increase Participation, Among Women of Black African and Black Caribbean Descent in the UK

**DOI:** 10.1002/pon.70093

**Published:** 2025-02-01

**Authors:** Anietie E. Aliu, Robert S. Kerrison, Afrodita Marcu

**Affiliations:** ^1^ School of Health Sciences University of Surrey Surrey UK

**Keywords:** Black African, Black Caribbean, Black women, breast cancer screening, ethnic inequalities, interventions, mammography, oncology, screening barriers, systematic review

## Abstract

**Background:**

Compared with the general population, Black African and Black Caribbean women, living in the UK, are less likely to participate in breast cancer screening.

**Aims:**

The aims of this review were to: (1) systematically describe the barriers to breast cancer screening, experienced by women of Black African and Black Caribbean descent, living in the UK and (2) identify which barriers interventions attempting to reduce inequalities have targeted, and whether they have been effective at increasing participation in breast cancer screening, among women of Black African and Black Caribbean descent.

**Methods:**

We conducted a mixed‐methods systematic review of primary research published in peer‐reviewed journals. Seven databases were searched, yielding eight articles for inclusion in the review.

**Results:**

Barriers to breast cancer screening participation were categorised into six analytical themes: ‘Understanding of, and concerns about, breast screening, the procedure and the reliability of test results’, ‘Misconceptions about, and lack of understanding of, the causes of breast cancer and personal risk’, ‘Emotional responses to cancer and screening’, ‘General barriers to accessing healthcare services (including breast screening)’, ‘Beliefs about cancer as a treatable disease’ and ‘Religious beliefs, cultural taboos and stigma’. These findings derive largely from studies with women who were not eligible for screening. Four studies testing interventions to promote uptake among ethnic minority groups were identified. None of them targeted Black African or Black Caribbean women, specifically; however, it was possible to extract data, for these groups, from the studies.

**Conclusion:**

Further research with women who are eligible for screening (but do not attend) is needed to verify the findings of this review. Studies targeting barriers experienced by Black women, particularly, are needed to reduce inequalities.

## 1 Introduction

Breast cancer is a leading cause of cancer‐related morbidity and mortality in the United Kingdom, with over 55,000 women diagnosed annually [1, 2]. While breast cancer survival has improved over recent years, with nearly 9 in 10 patients surviving 5 years or more [2], there are persistent ethnic and socioeconomic inequalities in breast cancer survival, which contribute to broader health inequalities [3, 4, 5]. These are most notable among women of Black African and Black Caribbean descent (hereafter referred to as Black women), who typically have poorer cancer experiences and outcomes [4, 6]. Indeed, compared with their White British counterparts, Black women are more likely to be diagnosed with breast cancer at an advanced stage and more likely to be diagnosed with aggressive forms of breast cancer (such as triple negative breast cancer), both of which are associated with poorer survival [3, 4, 6].

One potential avenue for reducing inequalities in breast cancer outcomes is through screening. Randomised controlled trials (RCTs) have shown that regular mammographic screening can significantly reduce mortality (from breast cancer) amongst women aged 50–70 years [7]. As a result, many European countries have implemented mammographic screening to improve breast cancer outcomes [8]. As with all screening, however, the extent to which the benefits of mammography are realised, and the extent to which they are equitable, is highly dependent on uptake. Studies consistently find that ethnic inequalities in breast screening persist among women from ethnic minority groups [9, 10, 11] and are most prominent among Black women [12, 13]. For example, a recent study by Kerrison et al. found that 44.95% of Black women attended a breast screening appointment, between March 2016 and September 2020, compared with 63.08%, 49.73%, 51.03% and 46.41% of women of White British, Asian, Mixed and Other ethnicity, respectively [10].

Previous reviews have synthesised the barriers to breast cancer screening among ethnic minority groups. Such reviews indicate a number of factors as barriers to breast screening, including stigma, religious beliefs, low risk perceptions and distrust in health professionals [14, 15]. However, these reviews have aggregated data from different ethnic minority groups, without making distinctions between them [16, 17, 18], despite wider research indicating that the barriers to healthcare vary between ethnic groups (e.g., due to differences in cultural attitudes and religious beliefs) [13, 19].

Earlier research has also tested the effectiveness of interventions to address barriers to breast cancer screening, experienced by women from ethnic minority backgrounds [10]. Community health education and health promotion workshops to raise awareness have been documented as being effective at increasing uptake in these populations [20]. Reports of improvement in knowledge and attendance from some ethnic groups, following targeted interventions, have also been reported in relation to South Asian and Somali women [9, 21]. To the best of our knowledge, there have been no reviews synthesising which interventions are effective for Black women, in particular. Such reviews are needed to inform policy designed to reduce inequalities in screening uptake and breast cancer outcomes. The present research is intended to address these gaps in the literature.

## 2 Methods

A systematic review was conducted to synthesise barriers to breast cancer screening, experienced by Black women, living in the UK. The study aimed to provide an overview of the evidence for the interventions designed to increase breast screening uptake.

### 2.1 Search Strategy

The search strategy was developed using the Population, Intervention, Comparison, and Outcome (PICO) framework [22], a mnemonic commonly used to help refine the research question, formulate eligibility criteria, and search strategies to retrieve studies for inclusion in reviews [23]. Supporting Information S1: Appendix 1 illustrates the application of the PICO framework and the corresponding search terms used in relation to each component.

We searched seven databases considered relevant repositories for peer reviewed articles on breast cancer screening, namely: MEDLINE, EMBASE, PsycINFO, PubMed, British Nursing Index, CINAHL (Cumulative Index to Nursing and Allied Health Literature) and Web of Science.

### 2.2 Selection Criteria

The search was restricted to studies published between 1988 (when the National Health Service Breast Screening Programme [NHSBSP] was introduced) and July 2023 (when the searches were conducted). Studies were eligible for inclusion if they met the following, pre‐specified, eligibility criteria: (1) reported the results of studies conducted in the UK; (2) were available in English Language and (3) investigated barriers or facilitators to breast screening or the use of interventions designed to promote uptake of breast cancer screening among Black women. Studies exploring barriers and facilitators to breast cancer screening, across multiple ethnic groups were eligible for inclusion, if they documented barriers or facilitators attributable to Black women. As a targeted population review, only those data that could be attributed to Black women were extracted and included in the analysis. Studies that focused on barriers and facilitators to (and interventions to promote the use of) breast cancer treatment, help‐seeking, symptom appraisal and self‐examination were excluded. Similarly, those not peer‐reviewed (i.e., ‘grey literature’) were also excluded. The full inclusion and exclusion criteria are documented in Supporting Information S1: Appendix 2.

The protocol for the review was registered with PROSPERO (CRD42023395839) and followed the Preferred Reporting Items for Systematic Reviews and Meta‐Analyses (PRISMA) guidelines [24]. The quality of included studies was assessed and described using the Mixed Method Appraisal Tool (MMAT) [25]. The tool was applied independently, by two authors (A.E.A. & R.S.K.), with disagreements arbitrated by a third author (A.M.). Similarly, two authors (A.E.A. & R.S.K.) independently assessed the articles at each stage of the selection process (i.e., title, abstract and full paper review). Here too, disagreements were discussed with, and arbitrated by, a third author (A.M.). Finally, the reference lists of papers passing full paper review were checked for additional relevant articles, which subsequently underwent abstract and full paper review, using the methods described for those identified through the database searches.

### 2.3 Data Synthesis

A mixed methods approach to data synthesis was adopted. In the first phase, qualitative data were analysed using thematic synthesis. The extracted data were coded, and themes were developed relating to barriers and facilitators of breast screening uptake. During the second phase, quantitative data that assessed barriers and facilitators of breast screening, and the effects of interventions to promote uptake, were categorised and described using narrative synthesis. In the final phase, findings from the thematic and narrative synthesis were triangulated to foster new insights into which barriers interventions may, or may not, address effectively.

Thematic synthesis is a method used to synthesise qualitative findings from qualitative research and allows researchers to identify patterns across a wide range of studies to provide a comprehensive and in‐depth understanding of the research topic [26]. It involves the development of themes through iterative coding of extracted data, such as participant verbatim quotes and original authors' themes and discussion. Narrative synthesis on the other hand was used to systematically summarise and interpret the findings from quantitative studies. It involves condensing and integrating the findings from individual studies to create a coherent and comprehensive narrative that addresses the research question or objective [27].

## 3 Results

### 3.1 Characteristics of Included Studies

**FIGURE 1:**
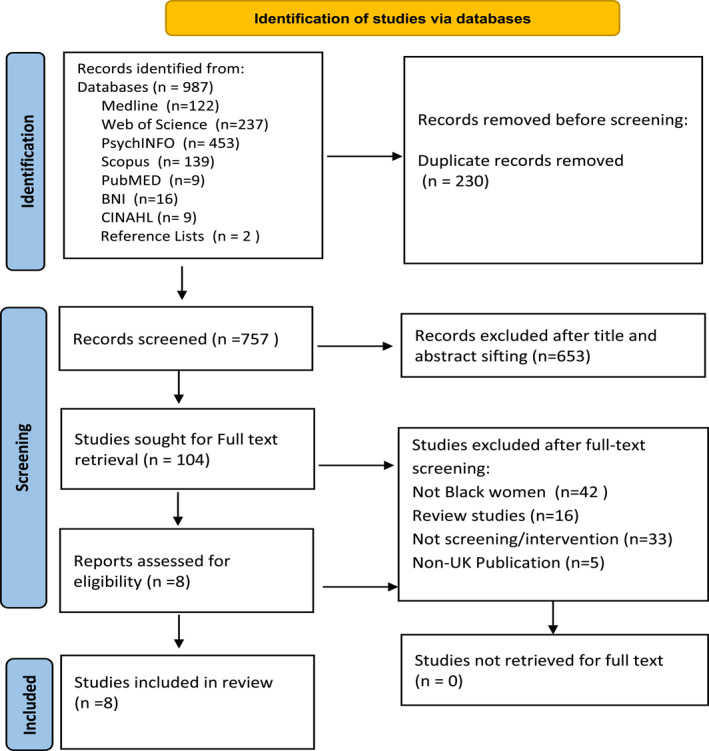
PRISMA 2020 flow diagram [24].

A total of 987 articles were identified from the database searches (see Figure 1). One‐hundred and four full text articles were eligible for evaluation. Of these, eight met the eligibility criteria and were included in the review. The sequence of study selection is illustrated in the PRISMA diagram [24] (see Figure 1).

Of the eight articles included in the review, four studies (3 qualitative, 1 quantitative) explored barriers and facilitators to breast cancer screening, and four intervention studies evaluated the effectiveness of interventions designed to improve breast screening participation (see Table 1 for a summary of the characteristics of included studies).

**TABLE 1:** Summary of the characteristics of included studies.

Publication	Aim	Design/methods	Sample size (overall/Black)	Age range of sample	% of sample who had attended screening	Analysis	Key findings
1. Bamidele et al. [14]	To understand screening uptake perceptions and factors that lead to low uptake of the breast cancer screening among Black African women in Luton, UK	Focus groups Qualitative	Black African women −25	38–62 38–44—11 (44%) 45–55—12 (48%) 56–65—2 (8%)	Not stated	Thematic analysis using framework approach	Limited awareness and information about breast cancer, reduce risk perception and anxiety from potential positive diagnosis. Study with Black African women from West African origin
2. Thomas, Saleem, and Abraham [28]	To describe factors that act as barriers to effective uptake of breast and cervical cancer screening services among black minority ethnic (BME) groups living in Brent and Harrow in the UK	Focus groups and telephone interviews. Qualitative	BAME—135 Blacks‐ African‐Caribbean (26, 18 females), West African (22, 12 females)	20–75 years	Not stated	Descriptive phenomenology with content analysis	Health and cultural beliefs, fear and lack of confidence in screening outcomes and relationship with health care professionals. Black participants identified as Black African and Caribbean women
3. Pfeffer [29]	Understanding why some women accept their invitation for mammography and others do not	Focus groups Qualitative	White women and BAME—146 Black Afro‐Caribbean 14 aged 35–49 = 9, aged 50–64 = 5	35–49 (younger/ineligible age) 50–64 years	Not stated	Candidacy and compliance	Cultural stereotypes, health negotiations. Black women in the BAME study identified as from Afro‐Caribbean descent
4. Atri et al. [13]	To determine if training general practice reception staff to contact non‐attenders could improve screening uptake, and whether women from different ethnic groups benefited equally	Controlled trial, randomised by general practice. Quantitative	All women—2064 Black 14% Indian 17%, White 31%, Pakistani 10%, Bangladesh 6%, Chinese 1%, other ethnic groups 4%, ethnic groups not reported 16%	50–64 years	Not stated	Multilevel logistic regression	Minimal but important improvement in screening uptake. Black women participants grouped as Blacks
5. Kernohan [20]	To provide information about breast and cervical cancer and related screening services to minority ethnic women and evaluate direct and indirect effect of uptake	Non‐controlled stratified sampling. Mixed method (survey and interview)	Ethnic minority women—1000 South Asian—670 African‐Caribbean—163, Eastern European 96 and other ethnic including Chinese—71	50–65 years	13% African‐Caribbean 20% all women	Descriptive & regression with intervention	Statistically significant increase in knowledge post community health education for the ethnic minority women including Black women from African/Caribbean descent
6. Offman et al. [12]	Telephone reminder intervention in London Newham in an area of high deprivation and ethnicity diversity	Observational study of planned intervention. Quantitative	From 29 Gp practices in Newham—10,928. Self‐reported ethnicity—Black (median 24%) White (24%), South Asian (37%) and mixed ethnicity (1%)	50–70 years	Not stated	Logistic regression	Significant decrease in the ‘adjusted log‐odds of attendance (ALOA)’ in practices with increasing proportions of Black patients (11%) decrease in odds of attendance per 10% increase in black population, CI 9%–16%, *p* < 0.001) Minimal response to screening in practice where black women were the majority
7. Bell et al. [21]	Interventions to improve uptake of breast screening in inner city Cardiff General Practices with ethnic minority lists	Observational study, with opportunistic intervention. Quantitative	Women invited—369 Non‐English speakers—218 (59.1%), English speakers—88 (47.1%) African‐Caribbean identified as English speakers, Somalian women as non‐English speakers	Not specified	Not stated	Descriptive & regression with intervention	Communication (language) was identified as a barrier for the Somalian women. The Somali women participants benefitted from link workers involvement
8. Barter‐Godfrey and Taket [11]	To understand women's breast screening behaviours	Cross‐sectional survey (Questionnaire) Quantitative	Females in Lambeth, Southwark, or Lewisham (*n* = 306) White British and Irish 73%, Black African 7%, Black Caribbean 10%, others 10%	50–64 years 50–54 years (39%), 55–59 years (34%), 60–64 years (27%)	Not stated	Chi square tests and binary logistics regression	Perception of low personal risk of developing breast cancer compared to White British and Irish and other BME women (chi square, *p* = 0.033)

The terms used to describe the study population differed across studies:

1. ‘Black African’ (Thomas, Saleem, and Abraham [28]; Barter‐Godfrey and Taket [11]; Bamidele et al. [14]);
2. ‘Black Caribbean’ (Barter‐Godfrey and Taket [11]; Thomas, Saleem, and Abraham [28]);
3. ‘Afro‐Caribbean/African’ (Bell et al. [21]; Kernohan [20]) and
4. ‘Blacks’ (Atri et al. [13]; Offman et al. [12]; Pfeffer [29]).

Five of the studies were conducted in London: two in Newham (Atri et al. [13]; Offman et al. [12]), two in Hackney, Brent and Harrow (Pfeffer [29]; Thomas, Saleem, and Abraham [28]) and one in Lambeth, Southwark, or Lewisham (Barter‐Godfrey and Taket [11]. The remaining three studies were conducted outside of London, covering Inner City Cardiff (Bell et al. [21]), Luton (Bamidele et al. [14]) and Bradford Communities (Kernohan [20]).

The participants in the reviewed studies were a mix of women eligible and ineligible for breast screening. For instance, a study in our review included more ineligible participants (*n* = 9, age 35–49) than women who were eligible for breast screening (*n* = 5, age 50–64) [29]. Similarly, another study incorporated both ineligible and eligible participants (*n* = 11, 38–44 years vs. *n* = 12, 45–55 years) [14]. Although the latter age classification is not in line with the criteria for screening eligibility (i.e., < 50 vs. > 50 years of age), it does give an indication that some participants were not eligible for screening. In another study, the classification of attendance behaviour implied that about 69% of the participants had attended breast screening with the intention to attend again in the future, while only 12% had never attended [11].

### 3.2 Thematic Synthesis of Key Barriers

Six key barriers were developed through the thematic synthesis of the qualitative evidence. An overview of the barriers is presented in Table 2. The following provides a detailed overview of the barriers, with illustrative quotes from the participants included in the studies.

**TABLE 2:** Analytical themes (key barriers).

1. Understanding of, and concerns about, breast screening, the procedure and the reliability of test results
2. Misconceptions about, and lack of understanding of, the causes of breast cancer and personal risk
3. Emotional responses to cancer and screening
4. General barriers to accessing healthcare services (including breast screening)
5. Beliefs about cancer as a treatable disease
6. Religious beliefs, cultural taboos and stigma

Barrier 1 Understanding of, and concerns about, breast screening, the procedure and the reliability of test results.

Studies reported that Black women often lacked understanding about the breast screening procedure, in particular, what the procedure involved and how it was performed.

…If we have better understanding of what the procedure is and what it entails, people might be more receptive….

[14]

Reduce awareness and knowledge of breast screening was also seen to impede engagement:

…yes…they wrote me a letter to come for breast screening, but I haven’t been there…two letters… now that you have talked about it, I think I would go and try….

[14]

Where Black women were aware of, and understood, the screening procedure, they reported concerns about the screening outcomes and the reliability of the screening test results. Indeed, concerns about ‘false positive’ and ‘false negative’ results were said to make people ‘lose confidence in the importance of screening’ [28]:

…cases of false positive or false negatives can also influence people’s decision to go for the screening, especially if someone is close to somebody who had experienced it….

[15]

Barrier 2 Misconceptions about, and lack of understanding of, the causes of breast cancer and personal risk.

Some studies reported that Black women often did not see themselves as being at risk of developing breast cancer, and therefore did not believe it was ‘for them’. In several instances, this was discussed in relation to their country of origin.

Yes, its [breast cancer] more common here than in Somalia.

[29]

I think it’s less common in our country than here or other civilized countries.

[29]

…they asked me to come about three months ago but I just ignored it, the other invitation I got, I just tore it…myself, I just believe if there is something like that in me, I will find it out myself….

[14]

Additionally, there were often misunderstandings about the causes of cancer, indicating poor understanding of the cause of breast cancer particularly, which may have contributed to the reduced risk perceptions.

…I think it’s an infection…I don’t know but it is not something that you can contract….

[14]

I’ve seen an African shop where they sell a cream that when people rub on their boobs, it makes it hard and firm, I think it can also cause cancer.

[14]

Barrier 3 Emotional responses to cancer and screening.

A number of studies found that Black women often expressed fear and anxiety about cancer and taking part in screening:

… and when I read the letter…I just tear the letter and threw it away. I did not tell anybody because I was so panicked….

[14]

Apprehension at the thought of receiving a breast cancer diagnosis, and about treatment, was also expressed. This was sometimes related to how others would perceive them, but also reflected stigma over loss of femininity and fear of partner abandonment, in the event of having breasts removed following a potential diagnosis:

More often than not, they take off the breast…the husband might want the breast…he might not be thinking of the health implication, he might be thinking so am going to have a wife without breast, so that might influence people going for the screening because he might be saying so if there is something, they will cut your breast off and for a woman that is feeling insecure in her home….

[14]

…misery, pain you know people going through that chemotherapy, I hear it is quite painful….

[14]

Barrier 4 General barriers to accessing healthcare services (including breast screening).

Reports of previous negative experiences with healthcare professionals and services were also found to influence the willingness of some Black women to attend health services. In one of the studies:

My GP, he sits like he is getting impatient that I am there, I am watching his body language, and I am asking ‘is it okay for me to be here are you sure’, he said ‘No, you go on…I am trying to think what help you really need here’ and I’m thinking don’t worry about it — I’m just wasting your time.

[28]

I go to the GP surgery and all he wants to do is to write a prescription, so now I don’t bother because what is the point of going.

[28]

Perceived poor communication from GP and experience of not being taken seriously were also reported to reduce intentions to take up screening [28]. For instance, the participants in Thomas, Saleem, and Abraham pointed to negative non‐verbal behaviour from the GPs, the practice receptionist and other health professionals in hospitals [28]. There was no explicit reference to previous negative experiences with breast screening (mammogram) or other cancer screening tests and services [14, 28].

In some cases, it was reported that a lack of access to screening often undermined the participation of Black women. This was due to the fact that some women were not registered with a GP, and thus did not receive invitations for screening, creating an unfair barrier to participation.

I know for those who are not registered with the GP, they might not get the information.

[14]

Barrier 5 Beliefs about breast cancer as a treatable disease.

The studies revealed conflicting beliefs about breast cancer as a curable disease. Fatalistic beliefs about cancer in general, as a death sentence, underpinned a culture of silence, signifying that cancer was not often discussed.

It’s really a death warrant because there is no cure for it, once you’re told that is it.

[14]

…most African people don’t like to talk about…that they have got cancer. They just see it as a taboo, in fact, I know a lot of people who do not even mention the word cancer. Before I had cancer I wouldn’t mention the word, I would just say that ‘c’ thing….

[28]

Where breast cancer was perceived to be curable, Black women indicated that they would take up screening, as they recognised that it promotes early diagnosis. The benefit of early diagnosis was described as a facilitator by some, who believed it subsequently made the disease more treatable:

I think the earlier its detected, the better the chances of survival and I think it also help to reduce the cost for the NHS because it is cheaper to screen than to treat….

[14]

If you have the option to have screening then at least you have a fighting chance you could go there, get screened and be cured. As much as you can be cured having cancer. It might not be your time to go.

[29]

We know that you can survive if it is caught early but one thing about cancer is that people don’t know much about cancer.

[14]

Barrier 6 Religious beliefs, cultural taboos, and stigma.

Many of the Black women, included in the reviewed studies, were reported to believe that they did not need to attend screening, as God would protect them.

He would find solution.

[28], 569

However, this was not true for all with religious beliefs, as some women were willing to compromise their religious ideals for health reasons and accept breast screening despite its invasive nature [29].

Because health sometimes demands exceptional measures, women are prepared to suspend the principles that govern their everyday lives in order to receive medical attention.

[28], 158

Superstitious beliefs, which presume breast cancer to be inflicted by spiritual powers, like black magic, or bewitchment, were also emphasised by some women, who reported that women would seek spiritual help, rather than attend breast screening or participate in early medical interventions [14]:

…it’s like oh my God who is that witch that has afflicted the person…so it’s…hindered some people to go for help, so instead of going to the hospital or something they start thinking of the mother‐in‐law or whoever…they don’t seek for medical help on time….

[14]

…most of the time when you have something like that, they will just attach it to witchcraft, evil spirit….

[14]

…that is African attitude and before you know it they will be moving from one spiritualist to another, spending money….

[14]

Finally, the fear of being stigmatised, if diagnosed with breast cancer, was a significant concern for women originating from Black African countries, in particular. They feared losing marriage prospects, or current partners [14].

…Sometimes they don’t want to lose their husbands, and they may be thinking if I go maybe he might leave me for another woman…I know some people who have run away from their marriages because the wife is maybe HIV‐positive or have breast cancer you know.

[14]

…let’s be honest, those of us that we are here you hear that the family that your daughter wants to marry into, they all have cancer, is that not a stigma? Is it not at the back of your mind that this family you want to marry into and all the women there they have cancer that what of the child you are going to have for them if she is a woman, she might have cancer as well….

[14]

### 3.3 Description of Barriers From the Quantitative Survey

One cross‐sectional survey of barriers and facilitators was identified. In their study, Barter‐Godfrey and Taket [11] explored personal reasons why women attend or do not attend screening. They included women from a range of ethnic minority groups, aged 50–64 years. The study observed that decision to attend breast screening was personal and was linked to individual attitudes. Women were more likely to attend breast screening if they reported screening was important to them. Furthermore, pain and anxiety were important factors that discouraged re‐attendance. Black women were, under‐represented in the survey (Black African: 7%; Black Caribbean: 10%; White British & White Irish: 73%). Nonetheless, the study found significant differences in perceived risk, specifically for Black women, who perceived their personal risk of developing breast cancer to be low, compared to the White British and other minority ethnic groups (Chi square, *p* = 0.033). Advice from the GP or health workers facilitated screening attendance (35%). On the other hand, most of the BME women (31%) declined to attend breast screening because of personal inconveniences. This finding corroborates those observed in the qualitative studies [11].

### 3.4 Narrative Synthesis of Interventions

Four quantitative studies testing the effectiveness of interventions were identified.

Atri et al. [13] conducted a randomised controlled trial, across 26 GP practices, to investigate whether uptake of screening could be improved by training reception staff to contact previous non‐attenders. The study results indicated that screening attendance was higher among Black women in the intervention group (telephone reminder) than among Black women in the control group (no reminder; 8% vs. 4%, respectively). Inferential comparisons were not possible, due to the small number of Black participants included in the research. The Black participants from different backgrounds (British, Caribbean and African), were treated as a single group in the analysis. The response from each group, consequently, was not differentiated. The barriers targeted or addressed were not specified [13].

Kernohan [20] evaluated the impact of a community health promotion intervention, employing a health education strategy (i.e., breast cancer and mammogram awareness workshops) to increase breast cancer knowledge and screening uptake in a non‐controlled, stratified sample, in Bradford. Six months post‐intervention, the study reported an increase in breast screening uptake by ‘African‐Caribbean’ women, compared with baseline (53.37% [*n* = 87] vs. 12.27% [*n* = 20]) [20].

Offman et al. [12] evaluated the effectiveness of a telephone reminder intervention to improve breast screening uptake, knowledge, and access, for ethnic minority groups. The study reported that, while the intervention was effective for South Asian women, with a significant increase in the Adjusted log‐odds of attendance (ALOA), in practices with higher proportions of registered South Asian patients (4% increase in odds of attendance per 10% increase in South Asian population, 95% CIs: 1%–7%, *p* = 0.003), there was a significant decrease in the odds of attendance in practices with higher proportions of Black women (11% decrease in odds of attendance per 10% increase in Black population, 95% CIs: 9% vs. 16%, *p* < 0.001) [12].

Finally, Bell et al. [21] conducted an observational study evaluating a multilingual screening information leaflet and GP endorsement letter, sent out to women 2 weeks before the screening invitation. Black African and Black Caribbean participants were categorised with the White British women as English speakers, while the Somali women were grouped as non‐English speakers [21]. The multilingual information leaflet was used to address language barriers. However, the majority of Somali women were unable to read English or Somali. Link workers were allocated to explain the information leaflet and help the women to make informed decision about attending the screening. Out of the 31 women visited by the link worker, 12 (41%) attended breast screening [21].

### 3.5 Triangulation of Thematic and Narrative Synthesis

Through the triangulation process, we sought to identify the barriers each intervention had attempted to address, and which interventions increased screening uptake. We began by summarising the barriers identified in this review (see Table 2). Followed by the interventions and the barriers they had attempted to address. We found that health promotion and breast awareness information were attempting to address: ‘Understanding of, and concerns about, the breast screening, the procedure and the reliability of test results’, while screening reminders to non‐attenders and prospective attenders aimed to tackle ‘General barriers to accessing healthcare services (including breast screening)’, by helping women to schedule appointments that are convenient. The intervention delivered through community health promotion appeared to lead to improvements in screening uptake among Black women [20, 21]. On the contrary, interventions addressing ‘General barriers to accessing healthcare services (including breast screening)’ through telephone reminders from GP practices did not appear to promote decisions leading to increased screening uptake among Black women, however, increase uptake was observed among South Asian women [12].

None of the interventions attempted to address other barriers identified in the thematic synthesis, such as emotional barriers (e.g., cancer fear), or cultural barriers (e.g., stigma).

## 4 Discussion

### 4.1 Summary of Key Findings

This mixed‐methods systematic review synthesised the barriers and facilitators to breast cancer screening reported by women of Black African and Black Caribbean descent. Four studies that highlighted barriers to breast screening, conducted in the UK, were included in the review and contributed to the synthesis of six overarching themes.

One of the key issues delineated from the literature, is that Black women often report uncertainty regarding the effectiveness and accuracy of breast screening. While there were gaps in knowledge about breast cancer and the screening procedure, lack of awareness about breast cancer risk factors perpetuated misconceptions of low personal risk of developing breast cancer [9, 28]. Similar barriers have been observed in cervical screening with the Black women [15] suggesting that the experience may not be limited to breast cancer screening.

Furthermore, emotional responses, including fear of potential positive diagnosis and anxiety about breast cancer treatment, loss of marriage partner or marriage prospect were consistent with emotional barriers to cervical and bowel screening, including cancer fear in general, experienced by Black women [10, 30] particularly those of Black African descent [14, 31]. This illustrates the importance of family networks for Black women, and a significant interplay between health, cultural, and social influences [15, 16].

Socio‐demographic and healthcare delivery barriers suggest that systematic issues such as accessibility to screening and lack of tailored communication strategies from health professionals can lead to mistrust. This mistrust is not only relevant to breast screening but also to other NHS cancer screening and health services, emphasising the need for culturally sensitive healthcare approaches [10, 15, 30].

Moreover, personal beliefs about breast cancer as either a treatable disease or a death sentence was a determining factor in breast screening uptake. Black women who believed that early detection leads to better treatment outcomes were likely to attend breast screening compared to those who believed it to be a death sentence [14]. Additionally, religious beliefs, cultural taboos and stigma played a crucial role in how breast cancer and screening were perceived, with some women feeling that discussing cancer is a taboo or against their religious beliefs. Cancer was often viewed as being inflicted by Black magic or from supernatural source [14]. This is supported by research on cancer fear and fatalism, for instance, Vrinten et al. found cancer stigma was higher amongst individuals from ethnic backgrounds [32].

Similar barriers are noted in early diagnosis of symptomatic breast cancer and cervical cancer, where excessive reliance on faith [13, 14, 33, 34, 35] and stigma surrounding cancer can prevent Black women from participating in screening [31, 36].

This study also reviewed interventions to address barriers and promote uptake among women of Black African and Black Caribbean descent. No studies targeting this population, specifically, were identified; however, four studies exploring the use of interventions to promote uptake among ethnic minority groups, broadly, were identified, many with data stratified by ethnicity.

From these four studies, we were able to delineate which interventions had been effective, and which barriers they successfully targeted. We found that community health education and awareness intervention attempted to address poor knowledge and understanding about cancer and concerns about breast screening while screening reminders to non‐attenders and prospective attenders aimed to tackle practical barriers to accessing healthcare services, including breast screening [12, 13]. For instance, Atri et al. telephone call reminder intervention to non‐attenders recorded an overall improvement in screening uptake in the intervention than the control group (8% vs. 4%); however, Black African and Caribbean women were grouped as Black participants and statistical analysis for each group were not specified [13]. Notably, there was observational evidence to support increase uptake of Breast screening by Black women using health education and community outreach [20].

### 4.2 Comparisons With the Previous Literature

Overall, the findings of this review are consistent with those reported in other studies exploring ethnic inequalities in cancer screening participation and help‐seeking behaviour. The following presents a comparison of the findings from this study with the extant literature.

This review identified misconceptions about the causes of breast cancer as a key barrier to breast screening. For example, in studies exploring ethnic inequalities to cervical screening, lack of knowledge was identified as an important barrier to screening uptake [15].

In addition, this study found that most Black women who live in the UK may not see themselves as being at risk of breast cancer. The relative low incidence of breast cancer among this population group may have facilitated these perceptions. However, this observation has also been reported in studies exploring ethnic inequalities in help‐seeking for breast cancer symptoms as well as cervical screening risk‐awareness which have included Black women [15, 33, 34, 37].

Importantly, the finding that emotional responses to cancer can act as a barrier to breast cancer screening is well established in the previous literature [14, 15, 19]. Women of Black African and Black Caribbean descent may experience emotional barriers more profoundly than their White British counterparts, due to cultural differences in perceptions of cancer for instance: Black women do not talk about cancer [14, 38] and cancer is pre‐determined and often linked to supernatural sources, the disclosure of diagnosis could result in being stigmatised [31, 36]. Our findings suggest they may experience a wider range of emotional barriers, including fear of positive diagnosis, family abandonment and stigmatisation [14, 15, 19].

This review has also identified the perception of breast cancer as a ‘death sentence’ as a prominent deterrent to screening uptake among Black women, particularly, women from African descent [11, 14]. This has been described as a barrier in several other studies [15, 31, 37]. Previous negative experiences with health professionals as potential disincentives to the uptake of health services has been previously documented [15, 36]. This study indicates that there is a communication gap between GPs and Black women. It suggests the need for culturally sensitive communication with GPs in breast screening and other health services to build trust. Lack of access to screening, due to non‐registration with a GP, was an important barrier to Black women due to immigration status. Similarly, the difficulty in making an appointment with the GP particularly among ethnic minority women were reported as barriers in help seeking for cancer symptoms [34].

Moreover, the present review found that some Black women who received the invitation failed to prioritise attending screening. This is a common theme throughout the barriers to screening literature where difficulties managing competing priorities and obtaining a convenient appointment are frequently identified as significant barriers to screening. These practical barriers were depicted among the wider BAME population and were linked to low socio‐economic status [37, 39].

Finally, the role of religious beliefs, cultural taboos and stigma are well established mechanisms underpinning inequalities in help‐seeking and screening participation. Previous research exploring differences in locus of control (how strongly people believe they have control over the situations and experiences that affect their lives), between ethnic groups, have found that women from ethnic minority groups score higher on measures of external locus of control (indicating they are less likely to believe they have control over the situations and experiences that affect their lives), and concluded that high religiousness may explain some of this variation (i.e., that it is ‘God's decision who lives and who dies', that ‘God's will cannot be defied through Western Medicine’, or that ‘God would save them’) [31, 35].

### 4.3 Implications for Policy and Future Research

The findings of this review have implications for future research. While research exploring barriers experienced by women of Black African and Black Caribbean descent have been published, these populations have been treated as a single group [19, 40]. Important cultural differences exist between women of African and Caribbean descent (e.g., women of African descent are more likely to report fear of being stigmatised by breast cancer than women of Caribbean descent). Future research should attempt to differentiate the cultural barriers experienced by each population. In addition, further research with women who are eligible for screening and/or do not attend screening is required to determine the ecological validity of the barriers and facilitators identified in this review, as the majority of participants included in the studies were either not eligible for screening or were attenders. The women not eligible for screening may experience different barriers to those reported by non‐attenders or screening eligible women [16].

Furthermore, the studies in this review were conducted in specific parts of England and Wales and may not reflect barriers experienced by women living in all countries of the UK. Likewise, studies of interventions were found (*n* = 4) but none explicitly focused on barriers experienced by Black women in the UK. Importantly, at present, it is not clear from the existing literature whether low attendance amongst ethnic minority groups is due to poor sampling strategies or ethnic minority groups not volunteering as study participants. Therefore, future research should implement better sampling strategies for recruiting Black African and Black Caribbean participants and find ways, perhaps targeted, for researchers to reach out to these populations [40, 41].

### 4.4 Strengths and Limitations

A strength of this review is the specificity of focusing on women of Black African and Black Caribbean descent, eliminating the crude groupings of Black, Asian and Minority Ethnic (BAME) group women together (which other studies have confirmed could mask salient differences) [19]. In addition, studies were drawn from peer‐reviewed literature, increasing the reliability of the data synthesised. Another strength of the systematic review lies in the methodological search of the peer‐reviewed literature, guided by PRISMA Framework.

The limitations to this review include: (1) some studies in this review were conducted with a mix of women eligible and non‐eligible for screening, meaning that some of the barriers may have been anticipated rather than directly experienced, thus limiting the validity of some of the findings; (2) the exclusion of grey literature, which may contain important insights and (3) exclusion of non‐English language papers (e.g., Welsh), which, again, may have provided useful insights.

## 5 Conclusion/Recommendations

This mixed‐methods systematic review identified a number of barriers to breast cancer screening experienced by women of Black African and Black Caribbean descent. It also found limited number of studies testing the effectiveness of interventions to promote breast cancer screening participation in these populations, as well as the barriers they attempted to address.

This study recommends more research representing Black African and Black Caribbean women across all countries of the UK including nuanced studies, differentiating between women of Black African and Black Caribbean heritage, as well as Black women from different Black African countries, who may experience different barriers to breast cancer screening, depending on their cultural background. Further research, including Black women who are eligible for screening, or who do not attend screening, is also required, as the present research has focussed largely on those who attend or are not eligible, to understand the differences in barriers experienced.

## Author Contributions

**Anietie E. Aliu:** conceptualization, methodology, data collection, formal analysis, writing – original draft, writing – review and editing. **Robert S. Kerrison:** conceptualization, methodology, data collection, formal analysis, writing – original draft, writing – review and editing, supervision. **Afrodita Marcu:** conceptualization, methodology, data collection, formal analysis, writing – original draft, writing – review and editing, supervision.

## Ethics Statement

The authors have nothing to report.

## Conflicts of Interest

The authors declare no conflicts of interest.

## Supporting information

Supporting Information S1

## Data Availability

Data available on request from the authors. The data that support the findings of this study are available from the corresponding author upon reasonable request.
