# Supplementary material for: A Systematic Review of Barriers to Breast Cancer Screening, and of Interventions Designed to Increase Participation, Among Women of Black African and Black Caribbean Descent in the UK
Source: Psychooncology. 2025 Feb 1;34(2):e70093. doi: 10.1002/pon.70093 (PMC11786783; doi:10.1002/pon.70093)
Supplement: Supplementary file 1 — Supporting Information S1 [file PON-34-e70093-s001.docx]

| **Appendix 1:** PICO SEARCH TERMS/ SEARCH STRING | |
| --- | --- |
| **Population (“Women of Black African of Black Caribbean descent”): “Black women”, “Black ethnic minority”, “Black African women”, “Black Caribbean women”.**  **Context (“United Kingdom”): “United Kingdom”, “England”, “Scotland”, “Northern Ireland”, “Wales”, “British”.**  **Intervention (“Breast cancer screening”): “Breast screening”, “mammography”, “breast neoplasm”, “early detection”, “early diagnosis”, “breast cancer screening”.**  **Outcome 1 (“Barriers and facilitators”): “uptake”, “motivation”, “barriers”, “facilitators”, “enablers”, “adherence”, “attendance”, “participation”, “attitudes”, “intentions”, “behaviour”, “awareness”.**  **Outcome 2 (“Interventions”): health education, patient education, health literacy.** | **TI (“Black wom#n” OR “Black ethnic minorit*” OR “Black African wom#n” OR “Black African*”) OR AB (“Black wom#n” OR “Black ethnic minorit*” OR “Black African wom#n” OR “Black African*” OR “Black Caribbean wom#n”)**  **MeSH (MH "Africa/EH") OR (MH "Blacks") OR (MH “Caribbean”)**  **Keywords: “Breast cancer screening” OR mammogra* OR “Breast screening” OR “breast neoplasam*” OR (breast AND (screening* OR “early detection” or “early diagnosis”))**  **MESH for Medline - (MH "Mammography") OR (MH "BreastNeoplasms/DG").**  **Keywords: (uptake OR motivat*OR ‘barriers OR adherence OR adherence OR knowledge OR facilitators OR attitude OR behavio#r OR awareness OR screen*)** |

| **Appendix 2.** Inclusion and exclusion criteria | |
| --- | --- |
| **Inclusion Criteria** | **Exclusion Criteria:** |
| - Qualitative, quantitative, and mixed method research - Studies that examine interventions related to breast cancer screening uptake by Black African/Black Caribbean women in the UK - Studies that investigate barriers or facilitators to breast screening uptake - For publications including other ethnicities, only data on women of Black African and Black Caribbean descent were extracted - Peer-reviewed literature published in English language. | - Studies reporting barriers or interventions for ethnic minorities other than Black African or Black Caribbean in the UK. - Studies of barriers or interventions to other cancer screening programmes. - Studies addressing breast cancer screening uptake among women of Black African and Black Caribbean descent in other countries. - Studies examining treatment of breast cancer. - Grey literature - Literatures published before 1988. |
